# Wilms’ Tumor Primary Cells Display Potent Immunoregulatory Properties on NK Cells and Macrophages

**DOI:** 10.3390/cancers13020224

**Published:** 2021-01-09

**Authors:** Piera Filomena Fiore, Paola Vacca, Nicola Tumino, Francesca Besi, Andrea Pelosi, Enrico Munari, Marcella Marconi, Ignazio Caruana, Vito Pistoia, Lorenzo Moretta, Bruno Azzarone

**Affiliations:** 1 Bambino Gesù Children’s Hospital, IRCCS, 00165 Rome, Italy; pierafilomena.fiore@opbg.net (P.F.F.); paola.vacca@opbg.net (P.V.); nicola.tumino@opbg.net (N.T.); francesca.besi@opbg.net (F.B.); andrea.pelosi@opbg.net (A.P.); vito.pistoia@opbg.net (V.P.); 2 Pathology Unit, Department of Molecular and Translational Medicine, University of Brescia, 25121 Brescia, Italy; enrico.munari@unibs.it; 3 Department of Pathology, IRCCS Sacro Cuore Don Calabria, Negrar, 37024 Verona, Italy; marcella.marconi@sacrocuore.it; 4 Department of Paediatric Haematology, Oncology and Stem Cell Transplantation University Children’s Hospital of Würzburg, 97080 Würzburg, Germany; Caruana_I@ukw.de

**Keywords:** Wilm’s tumor, NK cells, macrophages, tumor microenvironment

## Abstract

**Simple Summary:**

Wilms’ tumor (WT) is the most common childhood renal tumor accounting for approximately 7% of childhood malignancies. The overall survival rate for patients with favorable histology is greater than 90% while the survival rate for patients with poor prognostic factors is around 50%. The current treatments consist in a combination of surgery and chemotherapy or radiotherapy in high risk patients. Such treatments are responsible for significant adverse effects requiring long-term monitoring. Thus, a main challenge in WT treatment is the development of novel therapeutic strategies to eliminate or minimize the adverse effects. The characterization of an immune environment could allow the identification of new therapeutic targets. Herein we studied the interaction between WT and innate immune cells, in particular NK cells and monocytes. Although WT are highly susceptible to NK-mediated lysis, the detection of immunoregulatory activity of WT tumor cells on NK cells and also on monocytes could offer novel cellular and molecular targets for an efficacious immunotherapy of WT.

**Abstract:**

The immune response plays a crucial defensive role in cancer growth and metastasis and is a promising target in different tumors. The role of the immune system in Wilm’s Tumor (WT), a common pediatric renal malignancy, is still to be explored. The characterization of the immune environment in WT could allow the identification of new therapeutic strategies for targeting possible inhibitory mechanisms and/or lowering toxicity of the current treatments. In this study, we stabilized four WT primary cultures expressing either a blastematous (CD56^+^/CD133^−^) or an epithelial (CD56^−^/CD133^+^) phenotype and investigated their interactions with innate immune cells, namely NK cells and monocytes. We show that cytokine-activated NK cells efficiently kill WT cells. However, after co-culture with WT primary cells, NK cells displayed an impaired cytotoxic activity, decreased production of IFNγ and expression of CD107a, DNAM-1 and NKp30. Analysis of the effects of the interaction between WT cells and monocytes revealed their polarization towards alternatively activated macrophages (M2) that, in turn, further impaired NK cell functions. In conclusion, we show that both WT blastematous and epithelial components may contribute directly and indirectly to a tumor immunosuppressive microenvironment that is likely to play a role in tumor progression.

## 1. Introduction

Wilms’ tumor (WT) is the most common renal cancer in children accounting for approximately 90% of all pediatric kidney tumor and 7% of childhood malignancies [1]. WT arises from abnormal retention of embryonal renal tissue which has failed to differentiate properly and is characterized by several mutations considered responsible for tumor development [2]. Classical WT contains three histological components including epithelial, stromal, and blastemal ones. Blastema is similar to metanephric mesenchyme and represents the less differentiated component. Blastema can give rise to all WT components and is the most aggressive one with an adverse prognosis [3,4]. Dekel’s group identified CD56 (NCAM1) and CD133 as markers of renal progenitor populations, informative for both nephrogenesis and tumorigenesis [5,6,7]. Regarding tumors, CD56^+^/CD133^−^ cells correspond to the undifferentiated WT blastema fraction characterized by expression of markers of mesenchymal phenotype. Instead, in the process of nephrogenesis, CD56^+^/CD133^+^ and CD56^−^/CD133^+^ cells represent early and late stages of epithelial differentiation. These subsets exhibit a restricted tumor initiation capacity and tumor self-renewal as compared to CD56^+^/CD133^−^ cells [7,8].

The overall survival for children with Wilms’ tumor has reached 90% and 75% for localized and for metastatic disease, respectively. However, WT patients with high-risk histology and/or tumor relapses have a survival rate of 50% [9]. Although the current treatments led to improvements in survival rates, they are not target-specific and are responsible for significant long-term side effects, including heart toxicity, renal failure, infertility, and development of secondary malignancies [10,11]. Thus, the current aims of therapy is to increase survival in patients with high-risk tumors and to decrease the side-effects of treatment. Accordingly, the identification of new specific targets is required to improve the clinical outcomes.

Innate immune responses are thought to play a crucial role in the control of cancer growth. However, tumor infiltrating immune cells may exert either antitumor activity or promote tumor progression [12,13]. Natural Killer (NK) cells play a major role in the control of cancer progression and metastatic spread, primarily by mediating tumor cell killing and releasing pro-inflammatory cytokines and chemokines [13,14,15].

Human NK cell function is regulated by an array of inhibitory receptors, such as the HLA-I-specific killer immunoglobulin-like receptors (KIRs) and CD94/NKG2A. The inhibitory signals generated by the engagement with their MHC class I ligands result in NK cell inactivation. The CD94/NKG2A heterodimer, specific for HLA-E, is expressed first during NK cell differentiation. Later, it may be co-expressed with KIR(s) which recognize allotypic determinants of HLA-A, while it is lost by the most mature NK cells which express KIR(s) only [16,17]. In a healthy environment, resting NK cells express HLA class I molecules that generate inhibitory signals via KIR or NKG2A, while, NK cell activation is dependent on an array of activating receptors and co-receptors, including natural cytotoxicity receptors (NCR, i.e., NKp46, NKp30, and NKp44), NKG2D, DNAM-1, and CD16, that interact with ligands overexpressed or expressed de novo on tumor cells. Since tumor cells may display HLA down-regulation, NK cells can be triggered and kill target cell [16,17].

Usually, in solid tumors different types of cells infiltrating the TME such as Cancer Associated Fibroblasts (CAF), Mesenchymal Stem cells (MSC), myeloid suppressive cells, T reg. are responsible for an array of immune evasion mechanisms targeting both innate and adaptive immune cells [18,19,20,21,22,23].

To counteract the inhibitory effect of TME, several strategies have been developed that boost the antitumor effector function of NK cells [24,25,26,27,28]. Sometimes, malignant cells themselves may contribute to NK cell impairment mainly by secreting immunosuppressive molecules such as TGFβ, PGE2, IDO catabolites, and lactic acid [29,30,31,32,33].

On the other hand, other innate immune cells, such as macrophages, often display a pro-tumoral activity. Macrophages are heterogeneous cells that may acquire opposite functional capabilities in response to different microenvironmental signals. Thus, they can differentiate into classical activated macrophages (M1) with a pro-inflammatory profile or in alternatively activated macrophages (M2) which drive immune regulation and tissue remodeling [34,35]. M2 macrophages can be polarized by several stimulatory factors, which include cytokines (IL-4, IL-10, and IL-13), glucocorticoids, immune complexes (IC) and can be further subdivided into different subsets based on the applied stimuli. Typical IL-4-induced M2, also named wound-healing macrophages express high levels of mannose receptor (MR, also called CD206) and they secrete immunoregulatory factors such as TGF-β. This subset inhibits, through TGFβ, the production of IFNγ by NK cells but do not affect their cytolytic activity. M^MSC^ are induced by contact with MSC, down-regulate the expression by NK cells of NKp44, CD69 and the production of IFNg through the release of IL-10 and TGF-β but do not modify the release of lytic granules [36]. Another M2 subset termed tumor-associated macrophages (TAMs) is induced by co-stimulation with TLR ligands and A2 adenosine receptor (A2R) agonists or by IL-6 [36]. In addition, cancer cells, undergoing EMT promote M2-like polarization of tumor-associated macrophages through MIR21 induced by the transcription factor Snail [37]. These cells are mainly characterized by high IL-10, TGF-β, and vascular endothelial growth factor (VEGF) and constitute the major inflammatory component in neoplastic tissue that can suppress the antitumor immunity and promote metastatic progression [37]. All together, these data show that the different M2 subsets display peculiar immunosuppressive capabilities that affect different molecular pathways, which in turn may regulate NK cell activation.

Similar to other tumors, both adaptive and innate immune cells may infiltrate WT [38,39,40]. However, the relationship between such immune infiltrate and WT cells has not been investigated. Indeed, analysis of the effect of antitumor immune response may be useful for identifying therapies more specific for tumor cells and decreasing the toxicity of the current treatments.

In this study, we investigated the interactions occurring between WT primary cells and NK cells or monocytes. We could demonstrate WT-mediated immune regulatory effects on NK cells and monocytes which offer interesting targets for novel WT treatments.

## 2. Results

### 2.1. Characterization of WT Cell Cultures

**Figure 1:**
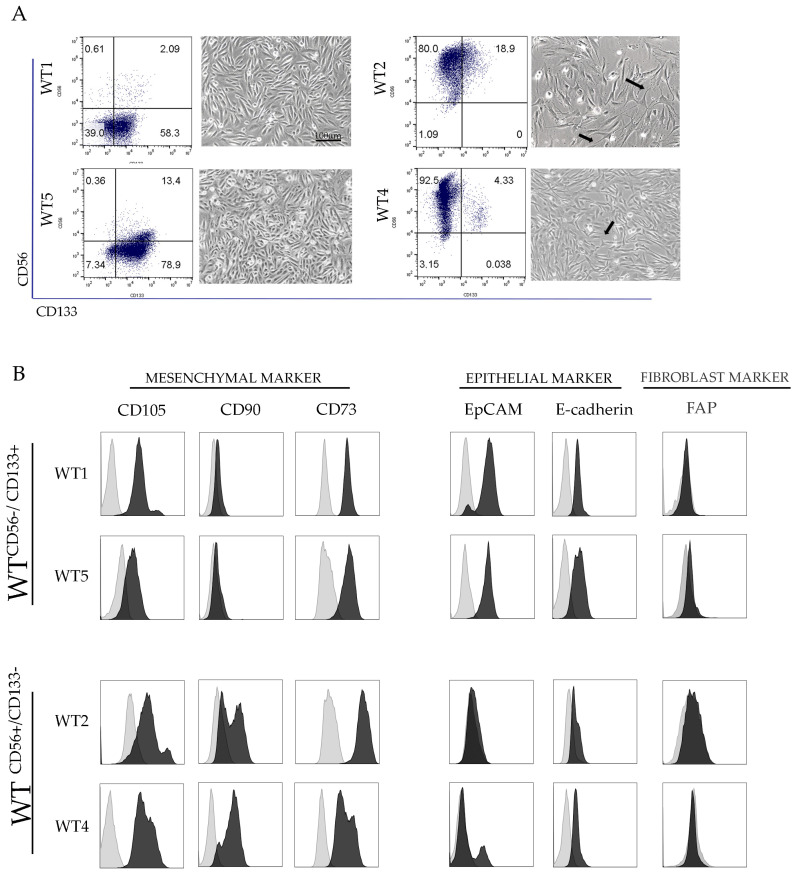
Phenotype characteristics of different Wilms’ tumor (WT) primary cultures. (**A**) Representative flow cytometry dot plot of CD56 and CD133 expression in WT primary cells and representative image of their morphology (arrows indicate cells displaying filamentous protrusions). (**B**) Surface expression of mesenchymal (CD105, CD73, CD90), epithelial markers (EpCAM and E-cadherin) or fibroblast marker (FAP) on different WT cells.

Primary WT cell cultures were established from tumors resected after preoperative chemotherapy from four different patients. Biopsies were obtained from primary tumor sites. We established four primary cell cultures termed WT1, WT2, WT4, and WT5. Then, we characterized the primary WT cell cultures on the basis of the cell surface expression of CD133 and CD56 following the classification previously described by Dekel’s group [5,6,7]. Flow cytometry showed that the majority of WT1 and WT5 cells (58.3% and 78.9%, respectively) expressed the CD56^−^/CD133^+^ phenotype. By contrast, the majority of WT2 and WT4 cells (80% and 92.5%, respectively) were CD56^+^/CD133^−^ (Figure 1A. The CD56^+^/CD133^−^ and CD56^−^/CD133^+^ WT cell cultures had different morphology. In particular, CD56^+^/CD133^−^ cell cultures displayed a fibroblast-like morphology with a bipolar organization, an elongated shape and several cell protrusions, while CD56^−^/CD133^+^ cells displayed an epithelial-like morphology with a polygonal shape. Subsequently, we further characterized the WT primary cells by investigating the surface expression of the main mesenchymal stem cell surface markers such as CD105, CD73, and CD90 [41] and epithelial markers such as EpCAM and E-cadherin. Cytometric analysis revealed that CD105 and CD73 were expressed in both CD56^+^/CD133^−^ and CD56^−^/CD133^+^ WT cells, while CD90 was expressed only in CD56^+^/CD133^−^ ones. Instead, the epithelial cell markers EpCAM and E-cadherin were expressed in CD56^−^/CD133^+^ but not in CD56^+^/CD133^−^ cell cultures (Figure 1B). In agreement with data of Dekel’s group, CD56^+^/CD133^−^ cells presented features similar to blastemal cells, while CD56^−^/CD133^+^ cells presented features similar to epithelial cells. None of the four WT primary cultures expressed the fibroblastic marker FPA, excluding the presence of tumor fibroblasts in the different WT primary cultures.

### 2.2. Detection of Infiltrating NK Cells within WT Tumor Mass by Immunoistochemistry

Cellular suspensions of WT biopsies are characterized by the presence of NK cells that infiltrate the tumor mass [38]. In order to determine which WT components were infiltrated by NK cells, we analyzed a Renal disease spectrum tissue array showing that NK cells, identified through the expression of the NKp46 marker, could be detected both within blastemal and epithelial WT tissues (Supplementary Figure S3).

### 2.3. WT Cells are Sensitive to Lysis Mediated by Activated NK Cells

**Figure 2:**
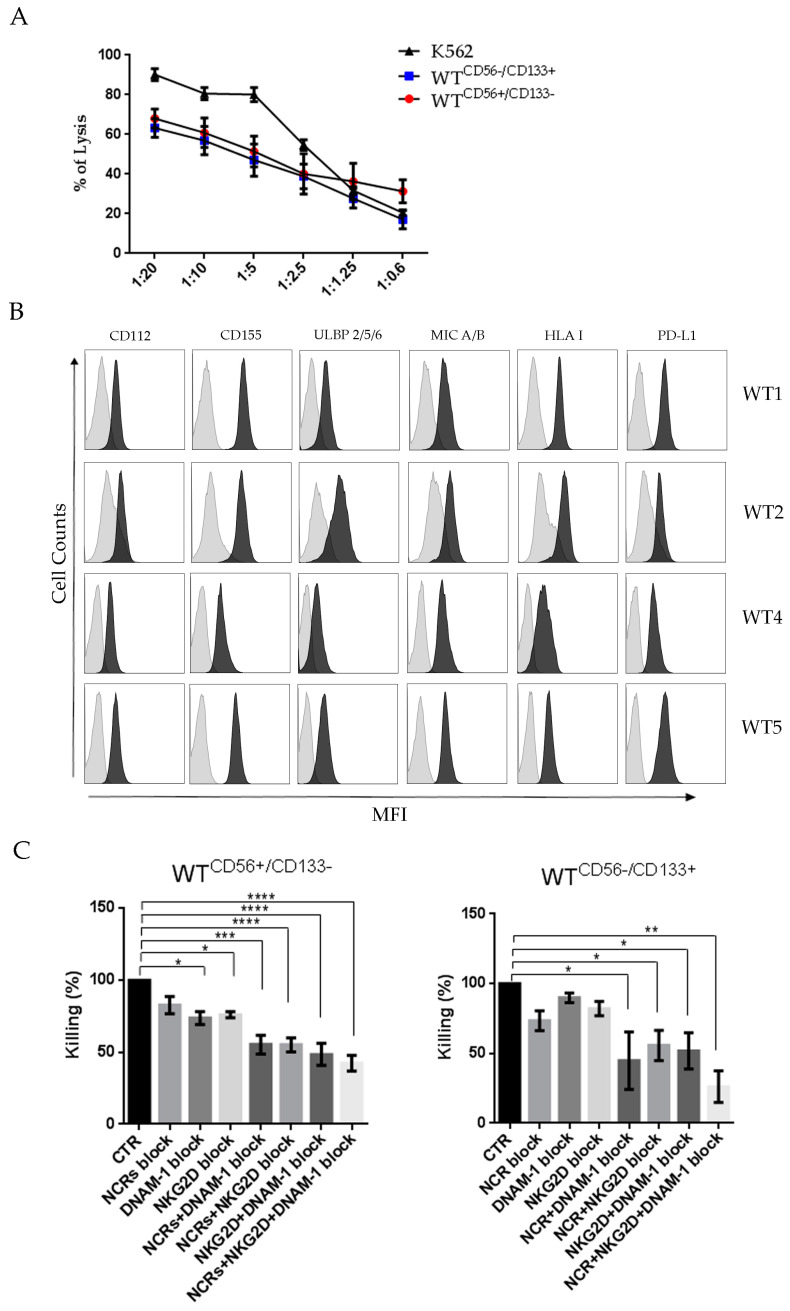
NK-mediated cytolysis of WT cell lines and ligand recognized by some activating and inhibitory receptors (**A**). The cytotoxicity assay of activated NK from healthy donors against WT cell lines. NK cells were incubated with WT cells for 4 h at the indicated E:T ratio. Data are the mean of four independent experiments for each WT cell line ± SEM. The NK susceptible K562 target cells were used as control. (**B**) Surface expression of NK receptor ligands: CD112, CD155, MIC A/B, ULBP 2/5/6, HLA I, and PDL-1 in a representative experiment. (**C**) Role of NCRs, DNAM-1, and NKG2D in killing WT cell lines. NK cells were incubated with WT cells (1:20 E:T ratio) in the absence or in the presence of saturating amounts (10 μg/mL) of anti-DNAM-1 and anti-NKG2D and multiple anti-NCRs blocking antibody. The graph shows the killing efficiency expressed as percentage of the killing observed with the control. The cytotoxicity assay data shown are obtained from single WT-CD56^+^/CD133^−^ and WT-CD56^−^/CD133^+^ cell cultures as target and activated NK cells purified from three healthy donors. Bar graphs are the mean ± SEM. *p*-values were calculated using one-way ANOVA with Dunnett’s multiple comparisons test (* *p* < 0.05, ** *p* < 0.01, *** *p* < 0.001, **** *p* < 0.0001).

WT is infiltrated by both adaptive and innate immune cells [39]. In order to investigate the relationship between NK cells and WT cells, we first examined the cytotoxicity of activated NK cells against WT cells. NK cells freshly isolated from peripheral blood (PB) of healthy donors were expanded in vitro with IL-2. They were incubated 4 h with WT cells and the percentage of WT cell lysis was detected by propidium iodide assay. As a control, NK cells were incubated also with the NK-sensitive K562 tumor cell line. As shown in Figure 2A, WT cells were sensitive to NK mediated lysis with 60 to 70% of killing at an Effector:Target (E:T) ratio of 20:1 (Figure 2A). This result indicates that WT cells are highly susceptible to NK-mediated lysis and that CD56^−^/CD133^+^ seem to be slightly more sensitive than CD56^+^/CD133^−^ WT cells. In view of these data, we further analyzed some informative ligands recognized by activating or inhibitory NK receptors. WT cell lines expressed CD112, CD155, ULBP2/5/6 and MIC A/B, i.e., the ligands of DNAM-1 and NKG2D activating NK receptors, respectively. Of note, they also expressed PD-L1, the ligand of the inhibitor checkpoint, PD1 (Figure 2B). Although WT cells expressed HLA class I, they were efficiently killed by NK cells, possibly reflecting levels of HLA expression non-sufficient to counteract the activating signals. To investigate the role of the Natural Cytotoxicity Receptors (NCRs), DNAM-1 and NKG2D in the NK-mediated killing of WT cells, cytolytic assays were performed in the absence or in the presence of anti-NCRs, anti-DNAM-1 or anti-NKG2D blocking monoclonal antibodies (mAb). In the presence of individual blocking mAb, a low decrease of the cytotoxicity was detected (Figure 2C). However, in most instances, NK-mediated killing of different tumors is known to be achieved by the combined effect of an array of activating receptors. Therefore, we further examined the effect of blocking simultaneously NCRs, DNAM-1 and NKG2D. A significant inhibition was detected, suggesting the idea that NK-mediated recognition and killing of WT cells requires the synergistic action of different activating receptors.

### 2.4. Inhibitory Effect of WT Cells on the Cytotoxic Activity of NK Cells in Co-Culture

**Figure 3:**
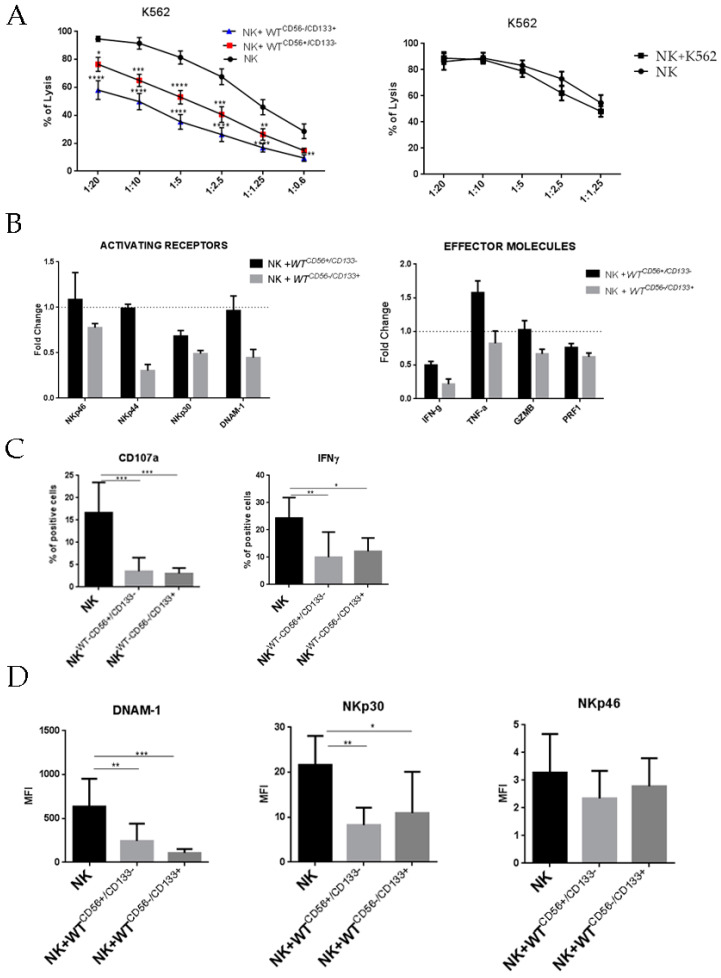
Evaluation of IL2 activated NK cell cytotoxic activity before and after co-culture with WT primary cultures (**A**) The cytotoxicity assay of NK cells from healthy donors cultured alone or with WT cell lines in the presence of IL2 on the left and alone or with K562 cells in presence of IL2 on the right. After six days, NK cells were incubated with K562 target cells for 4 h at the indicated E:T ratio. Data shown here are the average of four independent experiments for each WT cell line ± SEM. Statistical significance was determined by Student’s *t*-test (* *p* < 0.05, ** *p* < 0.01; *** *p* < 0.001; **** *p* < 0.0001). (**B**) mRNA fold change of activating receptors (NKp46, NKp44, NKp30, and DNAM-1) and effector molecules (IFNγ, TNFα, Granzyme B, and Perforine1). The results are the means ±SEM of three different experiments with different NK donors. (**C**) NK cell surface expression of CD107a and intracellular expression of IFN-γ after six days of culture alone or with WT cell lines under direct contact. The results are the mean ± SEM of three different experiments for each WT cell line. *p*-values were calculated using one-way ANOVA with Fisher’s LSD multiple comparisons test (* *p* < 0.05, ** *p* < 0.01, *** *p* < 0.001). (**D**) Surface expression of DNAM-1, NKp30 and NKp46 were measured by flow cytometry in NK cells on day six of culture with IL-2 alone or in presence of WT cells. Bar graphs show the mean fluorescence intensity (MFI) ratio between stained and unstained cells. The results are the means ± SEM of three different experiments for each WT cell line. *p*-values were calculated using one-way ANOVA with Fisher’s LSD multiple comparisons test (* *p* < 0.05, ** *p* < 0.01, *** *p* < 0.001).

In order to evaluate the possible immunosuppressive effect of WT on NK cell function, we investigated whether WT primary cultures could interfere with the effector function of PB-derived NK cells. To this end, freshly isolated NK cells and WT cells were co-cultured under direct contact or trans-wells conditions in the presence of IL2. At day six, NK cells were collected and tested for their cytolytic activity against K-562 (Figure 3A). Both WT primary cultures analyzed strongly inhibited the cytolytic activity of co-cultured NK cells, with a higher efficiency observed for WT CD56^−^/CD133^+^. In contrast, co-culture with K562 cells did not influence the cytotoxic potential of NK cells.

In Figure 3B we investigated in NK cells, by RT-PCR analysis, the modulation of the transcripts for activating receptors and cytokines induced by co-culture with WT primary cultures. The NKp30 transcript was down-regulated by both WT primary cultures, while the NKp44 and DNAM1 transcripts were down-modulated only after co-culture with WT CD56^−^/CD133^+^ cells. No modulation was observed for NKp46. Concerning the modulation of the cytokine transcripts we observed a down-modulation for the IFNγ transcript induced by both WT primary cultures. No significant modulation was observed for TNF-α, Granzyme B (GZMB) and Perforine1 (PRF1) transcripts.

In Figure 3C,D we investigated by flow cytometry the expression of CD107a, IFNγ and activating receptor. We observed a statistically significant decrease of the expression of the degranulation marker CD107a, of the IFNγ (Figure 3C) and of the activating receptor NKp30 and DNAM-1 (Figure 3D) induced by both WT primary cultures, which is consistent with an impairment of NK cell activation and impaired cytolytic function.

The impairment of cytolytic activity was detected only in NK cells cultured in direct contact with WT cells and not under trans-well condition (Figure S1A), suggesting that soluble factors released by WT cells do not play a major role in inhibition of NK cell function. Accordingly, the WT-mediated impairment of NK cytolytic activity was not prevented in presence of compound targeting IDO and PGE. The IDO catabolite Kynurenine and PGE are two soluble immuno-suppressive factors frequently released by tumor cells [42,43] (Figure S1B).

FACS histograms showing the modulation of the activating receptor expression have been added in supplementary Figure S3.

We also analyzed by flow cytometry the surface expression of the check-point molecules PD-1, TIM3, and TIGIT. Data show that PD-1 (Figure S4A) is almost undetectable at the surface of control NK cells and incubation with WT primary cultures does not modulate the expression of this check point on NK cells. By contrast, the expression of TIM is strongly and significantly decreased after co-culture with both WT primary cultures (Figure S4). Meanwhile, TIGIT is strongly expressed in control NK cells and decreases, even if not significantly, after co-culture with both WT primary cultures (Figure S4).

**Figure 4:**
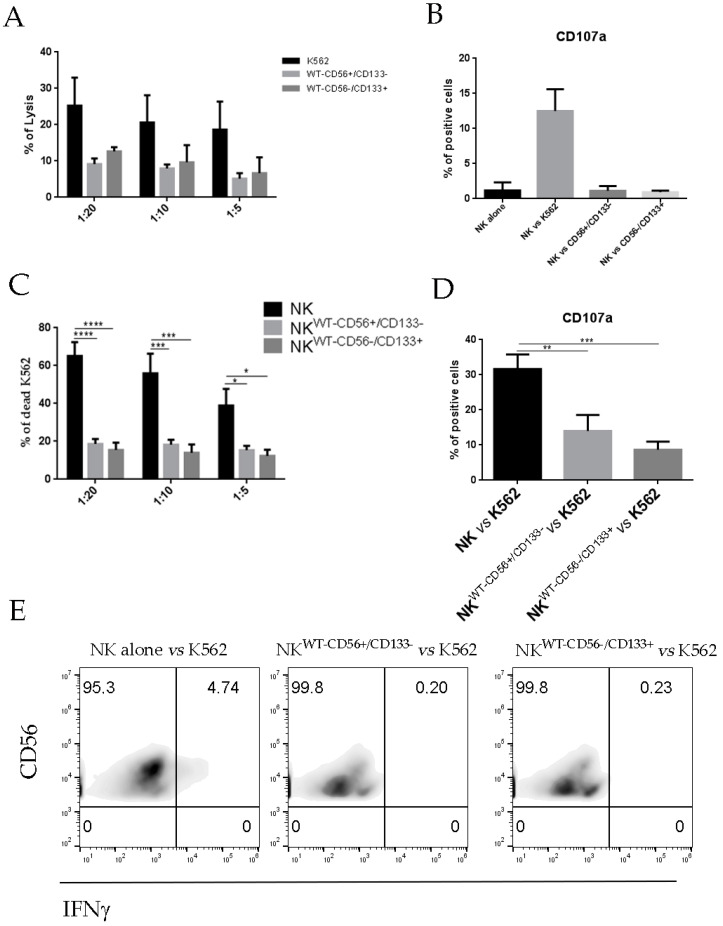
(**A**,**B**) Fresh NK cells were isolated from the peripheral blood of healthy donors and used as effector cells against different tumor cell line. K562 target cells were used as control. (**A**) Cytolytic activity of freshly isolated NK cells against different WT target cell line as indicated. Bar graphs represent the mean ± SEM (*n* = 3). (**B**) Percentage ± SEM of CD107a expression by freshly isolated NK cells against different WT target cell line as indicated (*n* = 4). (**C**–**E**) Freshly isolated NK cells were co-cultured alone or with WT tumor cell lines as indicated (WT 56^+^/133^−^ and WT56^−^/133^+^). After 48 h of conditioning, NK cells were tested as effector cells against K562 cell target. Percentage of (**C**) lyses ± SEM and (**D**) CD107a ± SEM expression using three different NK cell donors for each WT cell line. *p*-values were calculated using one-way ANOVA with Fisher’s LSD multiple comparisons test (* *p* < 0.05, ** *p* < 0.01, *** *p* < 0.001). (**E**) IFN-g production by NK cells upon co-culture with WT cell lines. One representative experiment out three was performed.

In addition, since NK cells infiltrating tumor TME are mostly non-activated NK cells, we performed the experiments described in Figure 2 and Figure 3 using resting NK cells, freshly isolated from healthy donors. As shown in Figure 4B, freshly isolated NK cells are not able to lyse WT CD56^+^CD133^−^ and WT CD56^−^CD133^+^ cells. Moreover, in Figure 4C,D we observed that 48 h of co-culture with WT primary cultures, induced in freshly isolated NK cells, resulted in an impairment of their lytic functions (lysis of K562 cells, and degranulation).

Since CD56 is a homotypic adhesion molecule and is expressed on human NK cells and on the CD56^+^/CD133^−^, but not on CD56^−^/CD133^+^ subset of WT cells, binding of NK cells to these different WT cell types could display different adhesion efficiency and therefore modify NK cell response. We have performed adhesion experiments using the adherent cell cytometry system Celigo. Analysis of Figure S5A shows that the differential expression of CD56 on WT primary cultures, does not influence the binding efficiency of NK cells to WT cells. Indeed, resting NK cells adhere with a similar efficiency both to blastemal and epithelial WT primary cultures. While, IL-2 activated NK cells display an increased adhesion efficiency without differences between blastemal and epithelial cells.

In Figure S5B, we analyzed at day six the viability of NK cells co-cultured or not with both WT primary cultures. Control NK cells display about 90% of viability and co-culture with both WT primary cultures does not significantly modify their viability.

### 2.5. WT Cells Modulate Monocyte Polarization

In these experiments, we investigated the potential ability of WT to modulate the function of other cells of the innate immunity known to play a role in tumor control/escape, we studied the interactions between WT cells and monocyte/macrophages.

**Figure 5:**
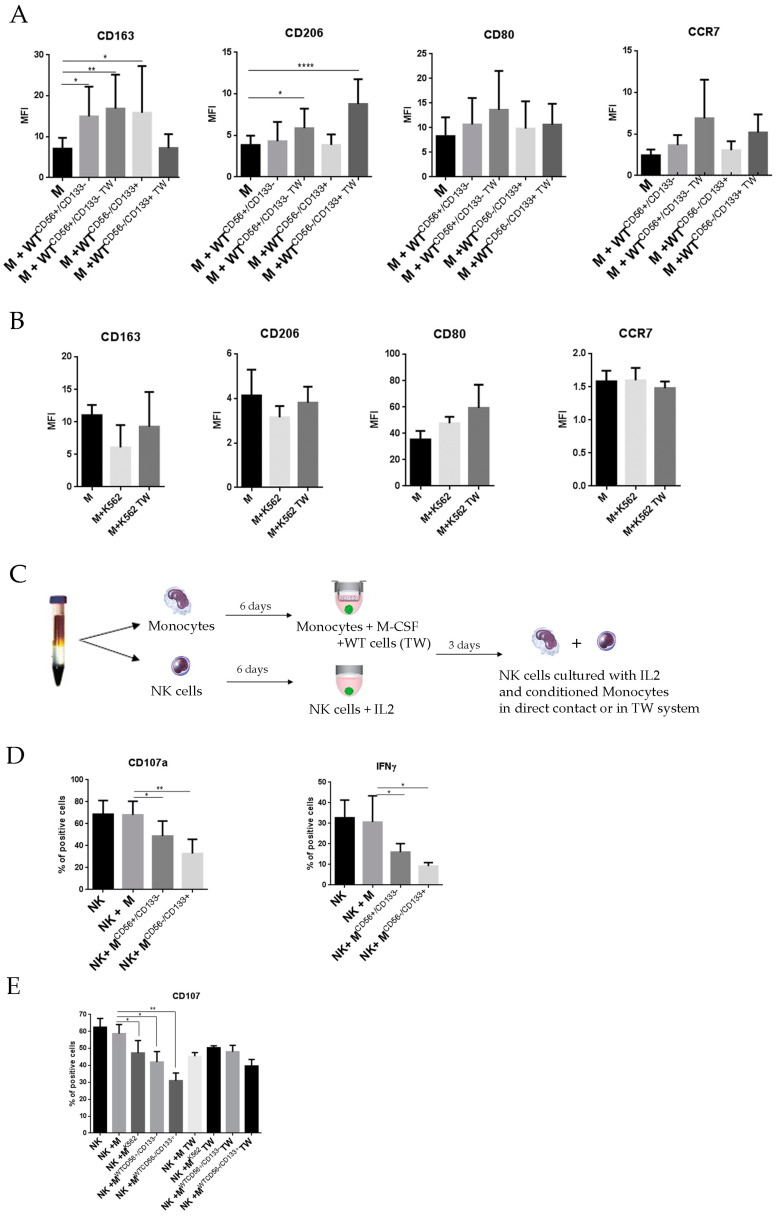
Effect of WT cells on monocyte polarization. (**A**,**B**) Surface expression of CD163, CD206, CD80, and CCR7 were measured by flow cytometry in macrophages at day six of culture with M-CSF alone or in the presence of WT cells (**A**) or in presence of K562 cells as control (**B**). Co-cultures were performed either in direct contact or in a tran-swell system (TW). Bar graphs show the mean fluorescence intensity (MFI) ratio between stained and unstained cells. The results are the means ± SEM of five different experiments for each WT cell line. *p*-values were calculated using one-way ANOVA with uncorrected Fisher’s LSD multiple comparisons test (* *p* < 0.05, ** *p* < 0.01). (**C**) Representation of the experimental design: monocytes were cultured with M-CSF in presence or absence of trans-wells insert with WT cells while autologous NK cells were cultured with IL-2. After six days, the trans-wells with WT cells were removed and monocytes were incubated with autologous NK cells for three days either in direct contact or in a trans-well system (TW). (**D**) CD107a expression and IFNγ production of NK cells after three days of co-culture with macrophages. The percentage of positive cells for CD107a and IFNγ was measured by flow cytometry. The results are the means ± SEM of three different experiments for each WT cell line and for K562 cells. *p*-values were calculated using one-way ANOVA with Fisher’s LSD multiple comparisons test (* *p* < 0.05). (**E**) CD107a expression of NK cells after three days of co-culture with macrophages in direct contact or in trans-well system. The percentage of positive cells for CD107a was measured by flow cytometry. The results are the means ± SEM of three different experiments for each condition. *p*-values were calculated using one-way ANOVA with Fisher’s LSD multiple comparisons test (* *p* < 0.05).

**Figure 6:**
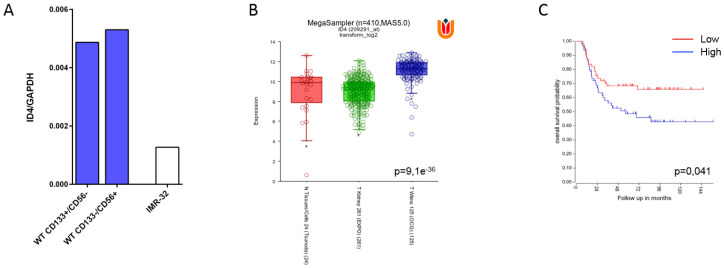
(**A**) ID4 expression measured by qRT-PCR in two Wilm’s tumor primary co-cultures with CD133^+^/CD56^−^ or CD133^−^/CD56^+^ phenotype. ID4 expression in the human neuroblastoma cell line IMR-32, which does not exhibit immunosuppressive effects on NK cells, is also shown. Data are expressed as 2-ΔCt with respect to GAPDH expression, used as endogenous control. (**B**) Gene expression analysis performed on R2 public dataset comparing the expression of ID4 between normal tissues (control, in red), kidney tumors (green) and Wilm’s tumors (blue). (**C**) Overall survival of patients with Wilm’s tumors included in the OCG study based on the expression of ID4. The analysis was performed on R2 public dataset using median as cutoff (124 patients, 62 low, and 62 high).

Therefore, monocytes were isolated from the PB of healthy donors and cultured with M-CSF and WT cells either in direct contact or in a trans-well system with WT cells in the lower well and monocytes in the upper well. After six days, the phenotype was analyzed by flow cytometry using CD80 and CCR7 as markers of monocyte polarization into classically activated macrophages (M1) and CD163 and CD206 as markers of polarization into alternatively activated macrophages (M2) (34,35). As shown in Figure 6A, monocytes cultured either in the absence or in the presence of WT cells displayed similar levels of CD80 and CCR7, thus suggesting the lack of M1-like polarization. By contrast, the expression of CD163 was higher in monocytes cultured with WT cells as compared to monocytes cultured alone and the expression of CD206 was higher in monocytes cultured with WT cells only under trans-well conditions. The up-regulation of CD163 and CD206 indicate that the WT cells may induce a M2-like polarization in co-cultured macrophages. We did not observe differences between monocytes cultured with CD56^+^/CD133^−^ or CD56^−^/CD133^+^ WT cells (Figure 5A). K562 cell line, used as the control, did not induce M2 polarization upon co-culture with PBMC monocytes (Figure 5B),

Then, we investigated whether WT primary cultures could express novel surface molecules that were able to induce M2 polarization. In this context, we analyzed the expression and role of ID4, a prognostic marker expressed by triple negative breast cancer cells that induces, upon co-culture, the M2 polarization of human PBMC monocytes [44]. Herein, we show by RT-qPCR analysis (Figure 6A) that ID4 transcript is much more strongly expressed in WT CD56^+^/CD133^−^ and WT CD56^−^/CD133^+^, in comparison with the control neuroblastoma cell line IMR32 that is lacking immunoregulatory properties (unpublished results).

In addition, we performed a gene-expression analysis querying the R2 public gene-expression dataset to assess the expression of ID4 in WT tumors. We found that ID4 is much more expressed in WT tumors than in normal renal tissue or renal cancers (Figure 6B). We then used the same dataset to evaluate the impact of the expression of ID4 on the patient’s overall survival (OS). A higher expression of ID4 was associated with a poorer clinical outcome (Figure 6C).

To test the immunosuppressive activity of M2 polarized macrophages induced by WT cells, these polarized macrophages were incubated, in cell-cell contact conditions, with autologous NK cells for three days, according to the timeline reported in Figure 5C. NK cells, cultured with WT conditioned monocytes in cell-cell contact conditions, showed a decreased expression of CD107a and IFNγ as compared to control NK cells (Figure 5D). We further investigated whether the inhibitory effect on NK cells was mediated by soluble factors. For this purpose, we used in vitro trans-well co-culture conditions in which the upper chamber contained activated NK cells that were separated, by a porous membrane allowing the passage of diffusible factors possibly released, by M2 cells seeded in the lower chamber. After three days of co-culture, no significant decrease of cytolytic activity (expression of CD107a) was detected in NK cells harvested from the upper chamber as compared to control NK cells (Figure 5E). These data suggest that the inhibition of the NK cells cytolytic function may not be the result of soluble inhibitory molecules released and diffused by M2 macrophages, or alternatively that the diffusible factors do not reach in the co-culture medium a concentration sufficient to induce biological effects, but rather requires M2-NK cell contact. The decrease of these effector molecules playing an important role in NK cell function indicates that WT cells can promote the generation of alternately activated macrophages which, in turn, exert immune-regulatory effects on NK cells.

## 3. Discussion

The interaction between tumors and the host’s immune system is crucial for the control of tumor growth and spread. Therefore, the knowledge of the cellular and molecular mechanisms involved in such interactions may help to develop new therapeutic approaches. Indeed, immunotherapy has successfully been applied to treat leukemias and, with less efficacy, solid tumors [45,46,47]. Very limited information is available regarding Wilm’s tumor [38,39]. An important tool to investigate mechanisms involved in WT/immune cell interactions is the availability of in vitro models reproducing such interactions.

So far, the establishment of primary WT cultures expressing a stable phenotype has been difficult and few cell lines expressing epithelial characteristics have been obtained, but no one exhibited blastemal morphology [48,49,50,51,52]. In this context, only the Dekel’s group was able to stabilize primary WT cells cultures exhibiting either spindle-shaped blastemal (CD56^+^/CD133^−^ or cobblestone epithelial (CD133^+^/CD56^−^) morphology and phenotypes. Blastemal cultures gave rise to serially cultured spheroids and exhibited CSC markers, but could not develop tumors in immunosuppressed mice [6]. The latter property was only detected in blastemal cultures derived from first-generation patient-derived–xenografts [8,53,54]. Finally, Royer-Pokora et al. stabilized in vitro primary WT1+ cultures exhibiting phenotypic and functional properties of Mesenchymal Stem Cells (MSC) [55], therefore these tumoral MSC should conceivably also display immunoregulatory properties [19].

In the present study, we were able to establish, from different patients, four primary WT cultures that were analyzed both for their phenotypic and functional characteristics. These studies were performed at low numbers of passages of cultured tumor cells (1–4) to avoid cell senescence or phenotypic and genotypic changes.

WT2 and WT4 cultures resulted highly enriched in cells expressing the CD56^+^/CD133^−^/CD90^+^/Epcam^−^/E-cadherin^−^ phenotype. According to Deckel’s classification, they represent blastemal cells. In contrast, WT1 and WT3 cultures expressed the CD56^−^/CD133^+^/CD90^−^/Epcam^+^/E-cadherin^+^ phenotype, thus representing epithelial cells [6,7,56]. Next, we investigated the effect of the interaction of different WT cells with cells of the innate immunity. In particular, we analyzed the ex vivo interaction between WT cells and NK cells showing by immunohistochemistry that NK cells, identified through the expression of the specific marker NKp46, infiltrate both blastemal and epithelial WT components. Thus, we tested both the susceptibility of WT cells to the cytolytic activity of NK cells as well as their possible immunoregulatory effect on NK cell function and on macrophage polarization. The NK cell activation and function are regulated by the integration of signals from activating and inhibitory receptors. We found that WT cells express several ligands for activating NK receptors, in particular the ligands for DNAM-1 (CD112 and 155) and NKG2D (MICA/B and ULBP2-5-6). Accordingly, we show that WT cells are efficiently killed by activated NK cells. Mab-mediated blocking of individual activating receptors NCRs, DNAM-1, and NKG2D partially reduces the NK-mediated killing while the simultaneous blocking of all such receptors induces a significant decrease of cytolytic activity.

It is well known that different tumors and their microenvironment may exert a strong inhibitory effect on immune cells, thus escaping the control of the immune system [18,19,20,21,22,23,24,25,26,27,28,29,30,31,32,33]. In this context, we investigated whether WT primary cultures were sensitive to NK cell mediated killing and could affect NK cell function upon co-culture. Both blastemal and epithelial WT primary cultures were killed by activated NK cells with an efficiency of about 70%, this however could mean that about 30% of the WT cells are resistant to direct NK-mediated killing. Based on these results, we reasoned that in the initial hours of the co-culture most WT cells would be killed, but that the surviving ones could represent a more aggressive subset able to impair, during the following days, NK cell functions. Indeed, we found, using both flow cytometric analysis and TaqMan Real Time PCR, that, after co-culture with WT cells, NK cells displayed a markedly decreased expression of the NCR molecules NKp30 and DNAM1, and an impaired cytolytic activity paralleled by a decreased expression of CD107a and production of IFNγ. In this context, according to previous studies, inhibition of IFNγ production may cause further immunologic effects such as inhibition of the development of T helper 1 (Th1) cells, maturation of DC cells and stimulation of tumor growth and metastatic process [57,58,59,60]. In addition, we also detected a strongly decreased expression of the check-point molecule TIM3. TIM 3 down-regulation induced by contact with WT primary cultures, is in agreement with results reporting that TIM-3 expression can be down-regulated on NK cells exposed to cancer cells [61]. Moreover, down-regulated TIM-3 expression correlated to lower cytotoxicity and lower interferon gamma (IFN-γ) expression [61] as we could observe in our experiments. On the other hand, high levels of TIGIT and its modest down-regulation associated to decreased NK cytolytic functions, is in agreement with literature data, since NK cells, exhibiting high TIGIT expression display poor cytolytic function, while NK cells exhibiting low TIGIT expression are characterized by very efficient cytolytic activity [62].

In the present study, co-culture experiments performed under trans-well conditions suggest that soluble factors secreted by WT cells may be not sufficient to impair NK cell effector function, which could be achieved only upon cell-cell contact. Moreover, addition of IDO and PGE2 inhibitors did not modify the cytotoxic potential of NK cells rendering unlikely the hypothesis that in the tumor microenvironment, paracrine loops of short-range interactions could play some role in impairing NK cells function. Since tumor-infiltrating NK cells are mostly “resting” non-activated, we also studied the interactions with WT primary cultures using freshly isolated resting NK cells. These cells, as expected, did not kill WT cells, but their functional impairment could be detected by their failure to kill K562 target cells after 48 h of co-culture with WT primary cultures. These data strengthen the concept that both resting NK cells co-cultured with WT cells, and tumor-infiltrating NK cells display functional anergy. In addition, it is indicated that although NK activation can induce lysis of WT cells, it does not rescue their cytotoxic activity after they have been in contact with WT cells, highlighting the immunosuppressive activity of blastemal and epithelial WT cells.

Several studies previously showed that the inhibition of NK function is dependent primarily on the down-regulation of activating receptors. This effect could be caused by the prolonged interaction with tumor cells expressing the activating receptors ligands. Indeed, we found that, after co-culture with WT cells, NK cells show a decreased expression of NKp30 and DNAM-1. Down-regulation of DNAM-1, induced by WT cells, was dependent on cell-cell contact. This effect has been reported to be per se sufficient to inhibit NK cell lytic function [63,64]. Thus, it is conceivable that this mechanism may by relevant also in favoring WT escape. In addition, WT cells express the immune checkpoint ligand PDL-1 that, in WT, is a biomarker of increased risk of therapy failure [65]. Our results are in line with recent papers reporting: (i) an increase, in several tumors, of infiltrating NK cells expressing PD1 as compared with circulating NK cells [23,66,67], (ii) the existence within WT tumors of an immune-engaged tumor microenvironment, characterized by infiltrating NK cells [38], and (iii) an elevated frequency of PD-L1 positive tumor cells in WT biopsies, predominantly in the blastema [65]. Therefore, it is likely that in vivo an intimate contact between blastemal, epithelial cells, and NK cells may lead to an impaired NK-mediated cytolytic activity while this cannot happen in vitro since PD1+ NK cells were not detected in the peripheral blood (PB) of healthy donors that we have examined.

Finally, we investigated the influence of WT cells on macrophage polarization. We found that WT cells could induce in vitro an M2-like polarization in monocytes isolated from PB, as shown by the induction of CD163 and CD206. In this context, we investigated whether our M2 macrophages expressed molecules able to induce M2 polarization and we focused our interest on ID4, a molecule recently detected on breast cancer cell lines, that behaves as a biomarker and induces polarization reprogramming of tumor infiltrating macrophages [44]. We have found that both WT primary cultures express high levels of ID4 transcripts. In addition, analysis of the R2 public data set shows that ID4 expression is much higher in WT tumors than in normal kidney or renal cancers and that its elevated expression in WT is associated with a poor clinical outcome. These data propose ID4 as a novel bio marker in WT tumor, possibly involved in the induction of the M2 polarization of human PBMC monocytes co-cultured with WT primary cultures.

Tumor-associated macrophages (TAM) are known to promote tumorigenesis supporting not only tumor cell survival and proliferation but also contributing to the induction of a suppressive TME. We found that WT-conditioned M2-like macrophages impair NK cell function primarily by interfering with the degranulation process (i.e., cytolytic activity) and IFNγ production. These effects were not observed under trans-well cultures but exclusively under M2-NK cell-contact conditions. However, based on these results, we cannot exclude the hypothesis that M2 macrophages may impair NK cell function through paracrine loops involving short range interactions.

Thus, we propose that the M2 polarized-macrophages that we originated constitute a novel M2 subset since they display an additional property (inhibition of degranulation) and act though cell-cell contact, differently from what was reported concerning IL-4-induced M2 and MMSC since these cells impair NK cytolytic functions trough IL-10 and TGFβ secretion [36]. By contrast, our data are in agreement with a recent paper showing that murine peritoneal, bone marrow, and tumor derived M2 inhibit NK lytic functions strictly by a cell-cell contact mechanism involving TGFβ [68]. This effect is likely mediated by TGFβ entrapped in the extracellular matrix [69] or in a membrane-bound form that is biologically active [70]. Thus, it is tempting to speculate that our M2 could act with a similar cell-cell-contact mechanism.

Since macrophage polarization is a reversible process, it may represent a promising target to interfere with tumor evasion mechanisms especially if associated with inhibition of check-point molecules on NK cells [71] in combination therapies [24,25,26,27]. This could be obtained: by Immune checkpoint suppression on NK, cells since WT primary cultures express ligands for both PD-1 and TIGIT rendering this therapy feasible [71]. Moreover, such clinical trials could possibly be improved, by associating for instance the IL-15-based harnessing of NK cells. This combination immunotherapy could represent an effective strategy, particularly in HLA-class I negative tumors that are ‘invisible’ to CD8+ T lymphocytes. Inhibition of M2 polarization could be achieved by: (i) targeting signaling pathways such as NF-κB and STAT3 [72], (ii) treating with drugs such as all-trans retinoic acid (ATRA) that, in other tumor models, inhibit M2 polarization [73], (iii) modifying the redox machinery using association of Auranofin and Vitamin C (two commonly available drugs) that, in a preclinical model, efficiently affected multiple redox pathways [74] or silenced the ID4 molecule.

Taken together, our data show that WT cells can affect NK cell antitumor activity both directly and indirectly by inducing macrophage polarization. These interactions are summarized in a scheme in Supplementary Figure S6. Interestingly, our data suggest that WT CD56^−^/CD133^+^ cells are both more susceptible to NK cells mediated killing and display a tendency to be more immunosuppressive for NK cells than WT CD56^+^/CD133^−^ cells. These data underline that the different WT components exhibit specific functions: blastemal cells possess higher aggressiveness, tumor initiation potential and self-renewal, while their immunoregulatory potential could be competence of blastemal subsets undergoing differentiation. On the other hand, epithelial cells, as tumor mass, would be a major component of the WT microenvironment displaying, together with the stromal component, extended immunoregulatory potential on NK cells. This property could be flattened in vitro by the use of K562 target cells that are extremely susceptible to NK cells, and could be highlighted employing targets such as NALM or RAJI cell lines that are more resistant to NK cells mediated killing.

In conclusion, we provide the first evidence that both blastemal and epithelial tumor components of WT, despite their high susceptibility to NK cells, can sharply compromise their function and act as well as on monocytes/macrophages. These cells, in turn, contribute to the development of an immunosuppressive TME further favoring tumor escape. These data offer a clue for the identification of novel cellular and molecular targets allowing to design novel strategies for the therapy of WT.

## 4. Materials and Methods

### 4.1. Cell Line Culture

K562 (human erythroblastoid cell line; ATCC) were cultured in RPMI 1640 (Euroclone, Milan, Italy) supplemented with 10% FBS (Euroclone), 1% penicillin/streptomycin (Euroclone) and 1% L-glutamine (Euroclone) in a humidified atmosphere with 5% CO_2_ at 37 °C.

### 4.2. Primary WT Cell Isolation and Culture

Primary WT cultures were obtained from WT biopsies resected after preoperative chemotherapy. Overflow human bioptic samples were obtained and treated in accordance with the policies and practices of the Institutional Etic Review Board of The OPBG Children Hospital Rome (# 1628/2018) and in accordance with the ethical principles stated in the Declaration of Helsinki. WT biopsies were washed in Dulbecco’s Phosphate-Buffered Saline (DPBS) containing Amoxicillina (0.6 mg/mL), Antibiotic Antimycotic Solution and 1% Pen–strep. WT cells were isolated directly from WT biopsies by using a mechanical digestion with gentleMACS Dissociator (Miltenyi, Bergish Galdbach, Germany) according to manufacturer indications and, then, using an enzymatic digested with 100 U/mL Collagenase II (Gibco) for 30 min at 37 °C. After digestion, the obtained cell suspension was washed with DPBS containing Amoxicillina, Antibiotic Antimycotic Solution, and Pen–strep, then filtered through 100, 70, and 40 µm cell strainers. Single cells suspensions of primary WT were resuspended in growth medium and plated in flasks. The growth medium was composed of Dulbecco’s Modified Eagle Medium/Nutrient Mixture F-12 (DMEM/F-12) (Gibco) supplemented with 1% Insulin-Transferrin-Selenium-Ethanolamine (ITS –X) (Thermofisher scientific, Waltham, MA, USA), 36 ng/mL Hydrocortisone (Sigma-Aldrich St. Louis, MO, USA), 4 pg/mL Triiodothyronine (T3) (Sigma-Aldrich), 10 ng/mL Putrescine (Sigma-Aldrich), 30 pg/mL Bovine Pituitary Extract (Sigma-Aldrich), 10 ng/mL Prostaglandin E1 (Sigma-Aldrich) and 10 ng/mL EGF (Invitogen). For passaging, the cells were detached using 0.05% trypsin/EDTA. After the initial seeding, cell confluence was reached within 7 to 10 days and the following subcultures were performed about twice a week. The cells necessary to perform the experiments were reached within four subcultures.

### 4.3. Purification of NK Cells

Fresh peripheral blood samples from healthy volunteers were provided by blood transfusion center of Bambino Gesù Pediatric Hospital. The Ethical Committee of OPBG approved the study (825/2014), which was conducted in accordance with the ethical principles stated in the Declaration of Helsinki. Peripheral blood mononuclear cells (PBMC) were separated by Ficoll-Hypaque density gradient centrifugation (Cederlane, Burlington, Ontario Canada). NK cells were isolated by RosetteSep human NK cell enrichment cocktail (Stem cell technologies SARL, Grenoble, France). For expansion and activation, purified NK cells were seeded in 96 well U bottom plate in MACS Medium^®^ (MIltenyi Biotec) with IL2 (600 U/mL Novartis, Basilea, Switzerland). Activated NK cells were used for cytotoxicity assays after two weeks of cultures.

### 4.4. Cytotoxicity Assays

Freshly isolated resting NK cells or two week-Activated NK cells with IL-2 were tested for their cytolytic activity in a flow-cytometric assay for NK-cell killing. WT or K-562 cells were used as targets for NK cell functional assays and different Effector:Target (E:T) ratios. WT or K-562 cells were stained with 5 μM Cell Tracker Green (CMFDA; Thermo-Fisher Scientific) and plated at 5000 cells/V-bottom 96-microwell plates. Target cells were incubated with NK cells at 37 °C at different E:T ratios. After 4 h of incubation propidium iodide (PI, Sigma-Aldrich) was added. Cells were acquired with the Beckman-Coulter Cytoflex-S flow cytometer and live target cells were identified as CMFDA+ PI− whereas dead target cells were CMFDA+ PI+. Percentage (%) of cell lysis was calculated as follows:

% cell lysis = ((% of dead cells cultured with NK) − (% of spontaneous lysis))/(100 − (% of spontaneous lysis) ) × 100

### 4.5. Co-Culture of NK and Target Cells

For co-culture experiments, freshly isolated resting NK cells or IL-2-activated NK cells (600 U/mL of IL-2) were cultured for two or six days, respectively (2.5 × 10^5^ cells/well) either alone or in the presence of WT primary cell cultures (0.5 × 10^5^ cells/well) in 24-wells plates in a 5:1 ratio. For trans-well culture conditions, we used an insert for 24-well plate with PET membrane bottom and pore size 0.4 µm (Sarstedt, Nümbrecht, Germany). Activated NK cells were seeded in the upper insert (2.5 × 10^5^ cells/well) and WT (cells/well) cells were seeded in the lower chamber (0.5 × 10^5^ cells/well) using a 5:1 ratio.

In the experiments with indoleamine 2,3dioxygenase (IDO) and Prostaglandin E2 (PGE2) synthesis inhibitors, in addition to 600 U/mL of IL-2 for 4 days, as previously described (26), we used: 1 mM of 1-methyl-tryptophan (Sigma-Aldrich, an IDO inhibitor capable to block kynurenine production) and 5 µM NS-398 (Sigma-Aldrich, an inhibitor of PGE2 synthesis). NK cells were collected and used as effectors in the cytotoxicity assays. K-562 cell line was used as control target for NK cell functional assays. For experiments with blocking antibodies, saturating amounts (10 μg/mL) of multiple anti-NCRs (anti-NKp46 (BAB281 clone, IgG1), anti-NKp30 (AZ20 clone, IgG1) and anti-NKp44 (Z231 clone, IgG1) mAbs), anti-DNAM-1 (F5 clone, IgM) and anti-NKG2D (BAT221 clone, IgG1) mAbs were added at the beginning of NK incubation with cell target.

### 4.6. Degranulation and Intracellular IFN-γ Staining

NK cell degranulation was determined by the cell surface expression of CD107a. NK cells, at the end of the co-culture with WT cells., NK cells were mixed with an equal number of K562 cells for 4 h in the presence of Monensin (BD Biosciences, GolgiStop) and APC-anti-CD107a mAb (Miltenyi Biotec).

The determination of IFNγ production was performed at the end of the co-culture period, the NK cells were stained with PC7-anti-CD56 (BD Biosciences) and then fixed and permeabilized with Fixation and Permeabilization Kit (BD Biosciences) and incubated with intracellular PE-anti- IFNγ mAb (Miltenyi Biotec).

### 4.7. Monocytes Polarization Induced by Primary WT Cell Cultures

Monocytes were selected from the PBMCs by positive magnetic selection (CD14 Microbeads, Milytenyi Biotec). Isolated monocytes were seeded for six days in low attachment 24-well plate in RPMI supplement with 100 ng/mL macrophage colony-stimulating factor (M-CSF) (Miltenyi Biotec). WT cells were added to monocytes at the ratio of 1:4 in direct cell contact or in trans-well for three days. For trans-well culture conditions, we used an insert for the 24-well plate with PET membrane bottom and pore size 0.4 µm (Sarstedt, Nümbrecht, Germany). Monocytes were seeded in the upper insert (4 × 10^5^ cells/well) and WT (10^5^ cells/well) cells were seeded in the lower chamber. Subsequently, the immunoregulatory potential of M2-polarized macrophages was determined analyzing CD107a expression and IFNγ production in homologous NK cells expanded for six days with IL-2 and then co-cultured for additional three days with control and M2-polarized macrophages. Co-culture was performed in direct cell contact or in trans-well culture conditions. In the latter setting, NK cells were seeded in the upper insert (4 × 10^5^ cells/well) and the control or M2-polarized macrophages (10^5^ cells/well) cells were seeded in the lower chamber.

### 4.8. Monoclonal Antibodies

For cytofluorimetric analysis, cells were stained with surface antibodies in PBS 5% FCS for 20 min at 4 °C. The following antibodies were used: CD133-APC, EpCAM -VioBlue, CD105-PE, CD90-VioBlue, IFNγ-PE, CD107a-APC, CCR7-FITC, CD163-PerCP-Vio700, CD206-VioBlue, HLA-DR-PerCP, CD80-APC (MIltenyi Biotec); CD56-PC7, NKp30-PE (Beckman Coulter); CD29-PE (ImmunoTools); CD146-PC7, CD73-FITC, NKp46-V450 (BD Biosciences); E-cadherin-APC (Thermo Fisher Scientific); N-cadherin-PE (Abcam); DNAM1-PE (Biolegend).

### 4.9. Cytofluorimetric Analysis

For detection of surface markers, WT or NK cells were stained with the fluorochrome-conjugated mAbs listed herein for 20 min at 4 °C. For detection of intracellular markers, NK cells were treated with the BD-Cytofix/cytoperm kit (BD-Biosciences, San Jose, CA, USA) according to manufacturer’s protocols and stained with indicated mAbs.

Cells were acquired with the Beckman-Coulter Cytoflex-S flow-cytometer (Beckman-Coulter, Brea, CA, USA). A minimum of 5000 events for each condition were acquired. The acquired data were analyzed with CytExpert-2.3 (Beckman-Coulter) and FlowJo v.10 software (BD-Biosciences).

Data were shown as mean fluorescence intensity ratio (MFI Ratio-mAb/unstained). Otherwise, the data were shown as Fold Change MFI, which represents the ratio between the sample stained with the selected mAb and the control stained with the selected mAb.

### 4.10. Plate Analysis with the Adherent Cell Cytometry System Celigo^®^

CD56^+^/CD133^+^ and CD56^−^/CD133^+^ cells were seeded at 3 × 10^5^ cells/well cells in 24 multi-well plates. Prior to plating, 72 h later, resting and activated NK cells were stained with Cell Tracker Green (Invitrogen) and seeded for 2 h on WT cells at a 1:5 ratio. Plates were read using the adherent cell cytometry system Celigo^®^ (Cyntellect Inc., San Diego, CA, USA) equipped with a brightfield, three fluorescent channels and a green filter for the Cell Tracker Green cytoplasmic dye. Gating parameters were adjusted for the green fluorescence channel to exclude background and other non-specific signals. The Celigo^®^ system provided a gross quantitative analysis for each fluorescence channel generating individual whole well images, including total count and average integrated green fluorescence intensity of gated events.

### 4.11. Immunohistochemistry

Membranous NKp46 expression was evaluated using standard immunohistochemistry. Staining was performed on 5-μm TMA sections (KD2084 Renal disease spectrum tissue array, US Biomax, Inc.) using a mouse monoclonal anti-NKp46 primary antibody (anti-NKp46/NCR1 195,314 R&D Systems for immunohistochemistry assay) and automated BenchMark ULTRA slide stainer, according to the manufacturer’s protocol. Sections were deparaffinized, rehydrated, and incubated for 32 min. The reaction was developed using 3–3-O-diaminobenzidine chromogen and counterstained with hematoxylin. Appropriate negative and positive controls were used.

### 4.12. Gene expression Analysis in NK Cells

For gene expression analysis in NK cells, we employed IL-2 activated NK cells before and after six days of co-culture with both WT primary cultures. Total RNA was reverse transcribed with random primers using Super Script IV first-strand synthesis system following manufacturer’s instructions (Thermo Fisher Scientific, Wilmington, DE, USA). Real Time PCR was performed by 384-wells TaqMan array microfluidic cards with a custom configuration for the detection of selected genes implicated in NK cell biology (Thermo Fisher Scientific, Wilmington, DE, USA). Briefly, 200 ng of cDNAs for each sample were mixed with an isovolume of TaqMan Advanced Master Mix 2X and loaded in 384-wells cards (100 ng/channel). Real time PCR were carried out on a QuantStudio 12 Flex instrument using thermal PCR cycling conditions suggested by the manufacturer (Applied Biosystems, Foster City, CA, USA). Data analysis was performed on Thermo Fisher Cloud with Design and Analysis New qPCR application (Thermo Fisher Scientific, Wilmington, DE, USA).

### 4.13. Quantitative Real Time Quantitative-PCR (RT-qPCR) Analysis

Total RNA extraction from WT cells was performed with miRNeasy mini kit with on-column DNase-I treatment following manufacturer’s protocol (Qiagen, Hilden, Germany). Five hundred ng of total RNA was reversely transcribed with random primers by using Super Script IV first-strand synthesis system following manufacturer’s instructions (Thermo-Fisher Scientific). Real time PCR were carried out in 20 µL of total volume in triplicate with TaqMan™ Fast Advanced Master Mix (Applied Biosystems, Foster City, CA, USA). The following TaqMan™ Gene Expression assays were used: ID4 (Hs02912975_g1) and GAPDH (Hs00266705_g1). Real time PCR were carried out on a QuantStudio 6 Flex instrument (Applied Biosystems, Foster City, CA, USA).

### 4.14. Statistical Analysis

Sample size used is indicated in the legend of each figure. Quantitative data are presented as means ± SD or means ± SEM. Statistical analysis was performed with Prism 6 (GraphPad Software, San Diego, Calif). Normality was tested with the Shapiro-Wilk test. Mann Whitney test, Student’s *t*-test, Dunnett’s, or Fisher’s LSD multiple comparisons test were used to determine statistical significance. Differences were considered significant for *p* < 0.05.

## 5. Conclusions

We show that Wilm’s tumor exhibits immunosuppressive properties on NK cells and monocytes, promoting an immunosuppressive microenvironment. We are convinced that these data may lead to the detection of novel cellular and molecular targets favoring the development of innovative immunotherapy protocols for Wilms’ Tumor.

## Supplementary Materials

The following are available online at https://www.mdpi.com/2072-6694/13/2/224/s1, Figure S1. (A) The cytotoxicity assay of NK cells from healthy donors cultured alone or with WT cell lines under trans-wells condition. After six days, NK cells were incubated with target cells K562 for 4 h at the indicated E:T ratio. Data shown here are the average of four independent experiments for each WT cell line ± SEM. (B) The cytotoxicity assay of NK cells from healthy donors cultured alone or with WT cell lines in direct contact and in the presence or absence of IDO and PGE2 inhibitors (1-MT and NS-398). After six days, NK cells were incubated with target cells K562 for 4 h at the indicated E:T ratio. Data shown here are the average of five independent experiments for each WT cell line ± SEM. Figure S2: Surface expression of CD69 and PD1 were measured by flow cytometry in NK cells on day six of culture with IL-2 alone or in presence of WT cells. Bar graphs show the mean fluorescence intensity (MFI) ratio between stained and unstained cells. The results are the means ± SD of three different experiments for each WT cell line. Figure S3: Surface expression of N-cadherin, CD29, CD146, and CD47 on different WT cells.
