# Supplementary material for: Wilms’ Tumor Primary Cells Display Potent Immunoregulatory Properties on NK Cells and Macrophages
Source: Cancers (Basel). 2021 Jan 9;13(2):224. doi: 10.3390/cancers13020224 (PMC7826641; doi:10.3390/cancers13020224)
Supplement: Supplementary file 1 [file cancers-13-00224-s001.pdf]

Supplementary

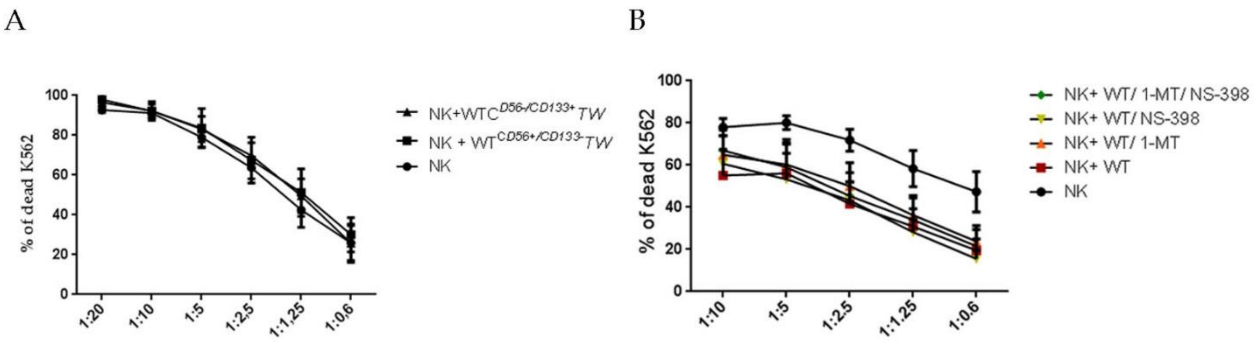

Figure S1. (**A**) The cytotoxicity assay of NK cells from healthy donors cultured alone or with WT cell lines under transwells condition (TW). After 6 days, NK cells were incubated with target cells K562 for 4 hours at the indicated E:T ratio. Data shown here are the average of four independent experiments for each WT cell line ± SEM. (**B)** The cytotoxicity assay of NK cells from healthy donors cultured alone or with WT cell lines in direct contact and in the presence or absence of IDO and PGE2 inhibitors (1-MT and NS-398). After 6 days, NK cells were incubated with target cells K562 for 4 hours at the indicated E:T ratio. Data are the average of five independent experiments for each WT cell line ± SEM

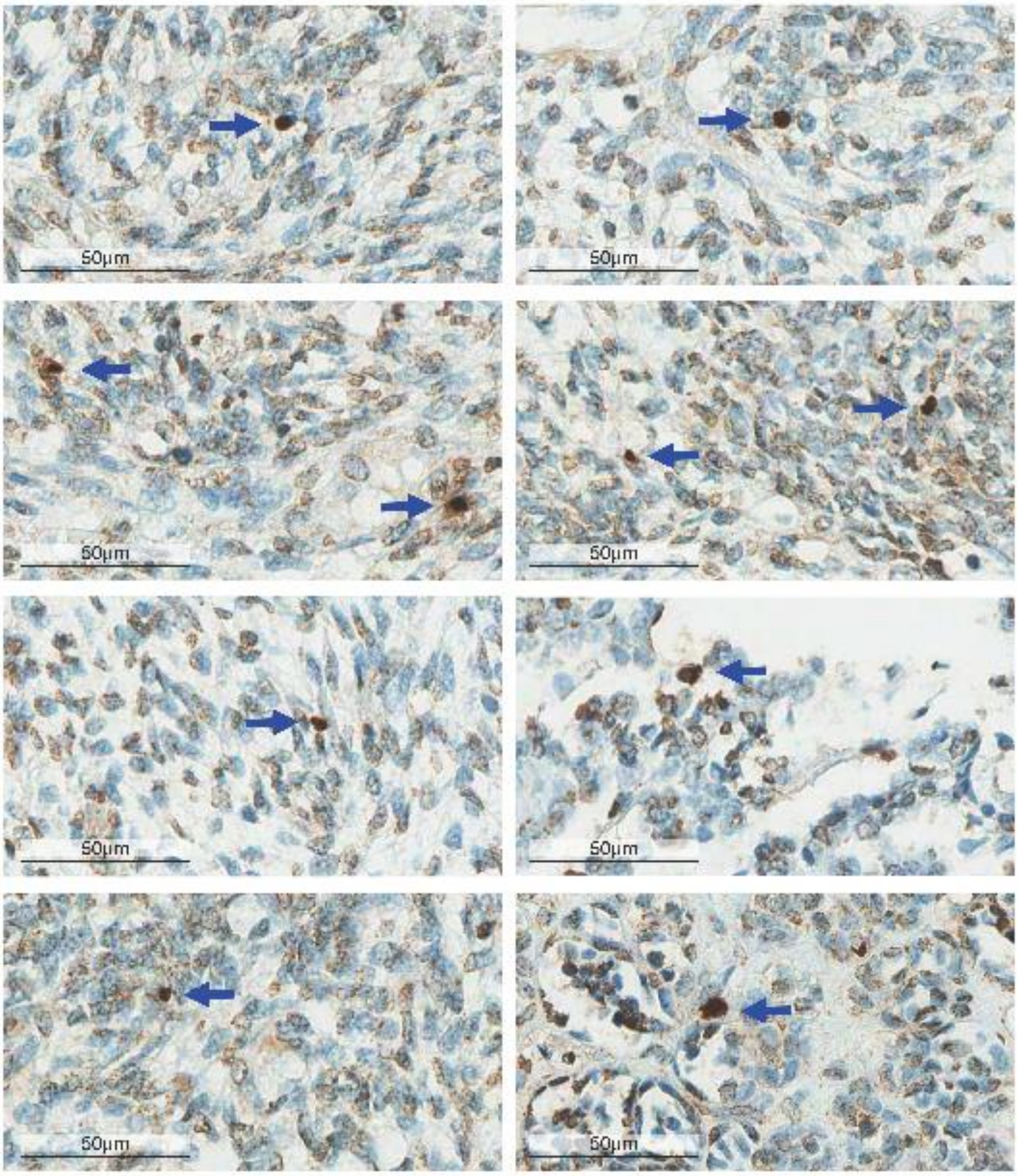

Figure S2. Detection of NKp46 positive cells in human WT tissue sections by IHC. Representative images of sections of 19 cases of WT tissue. Arrows indicate infiltrating NK cells.

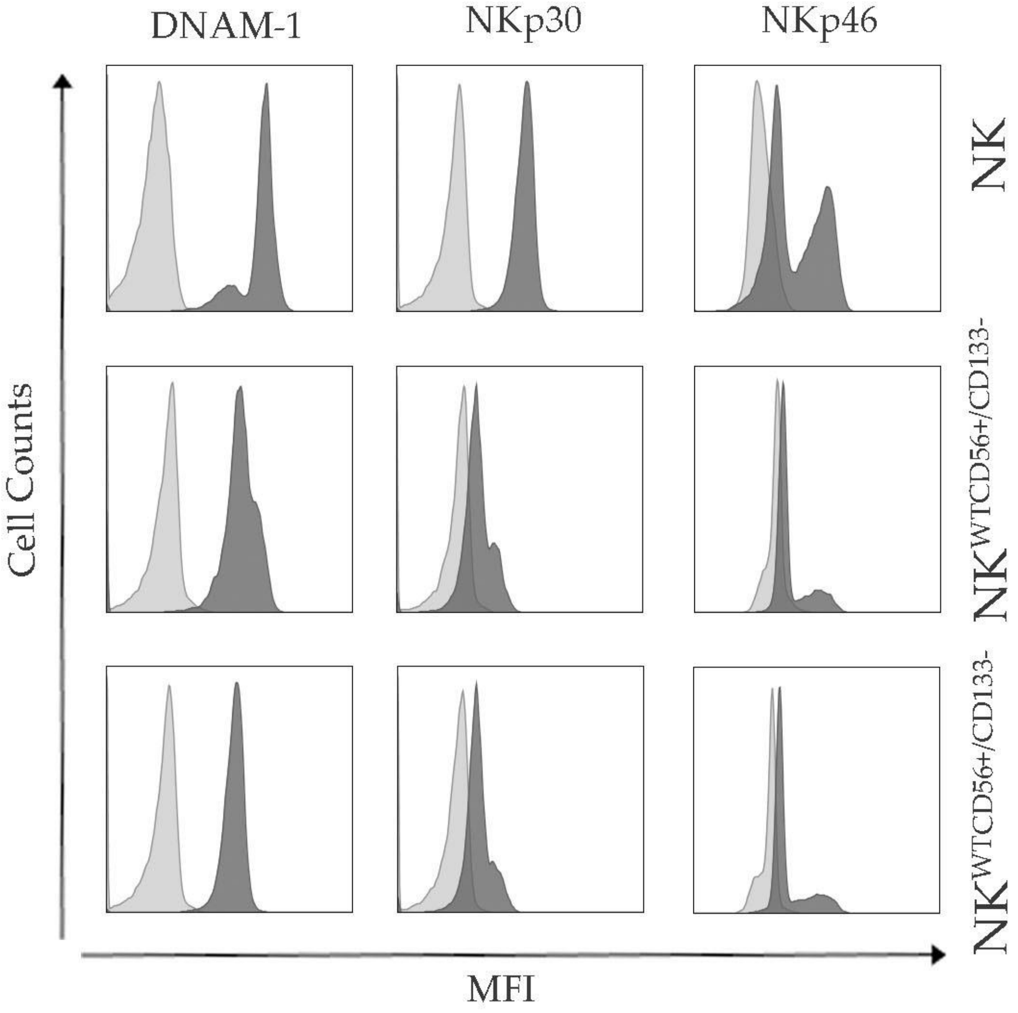

Figure S3. Surface expression of DNAM-1, NKp30 e NKp46 was measured by flow cytometry in IL-2 activated NK cells cultured alone or with WT cells for 6 days. One representative experiment out of 4 performed was presented as histograms.

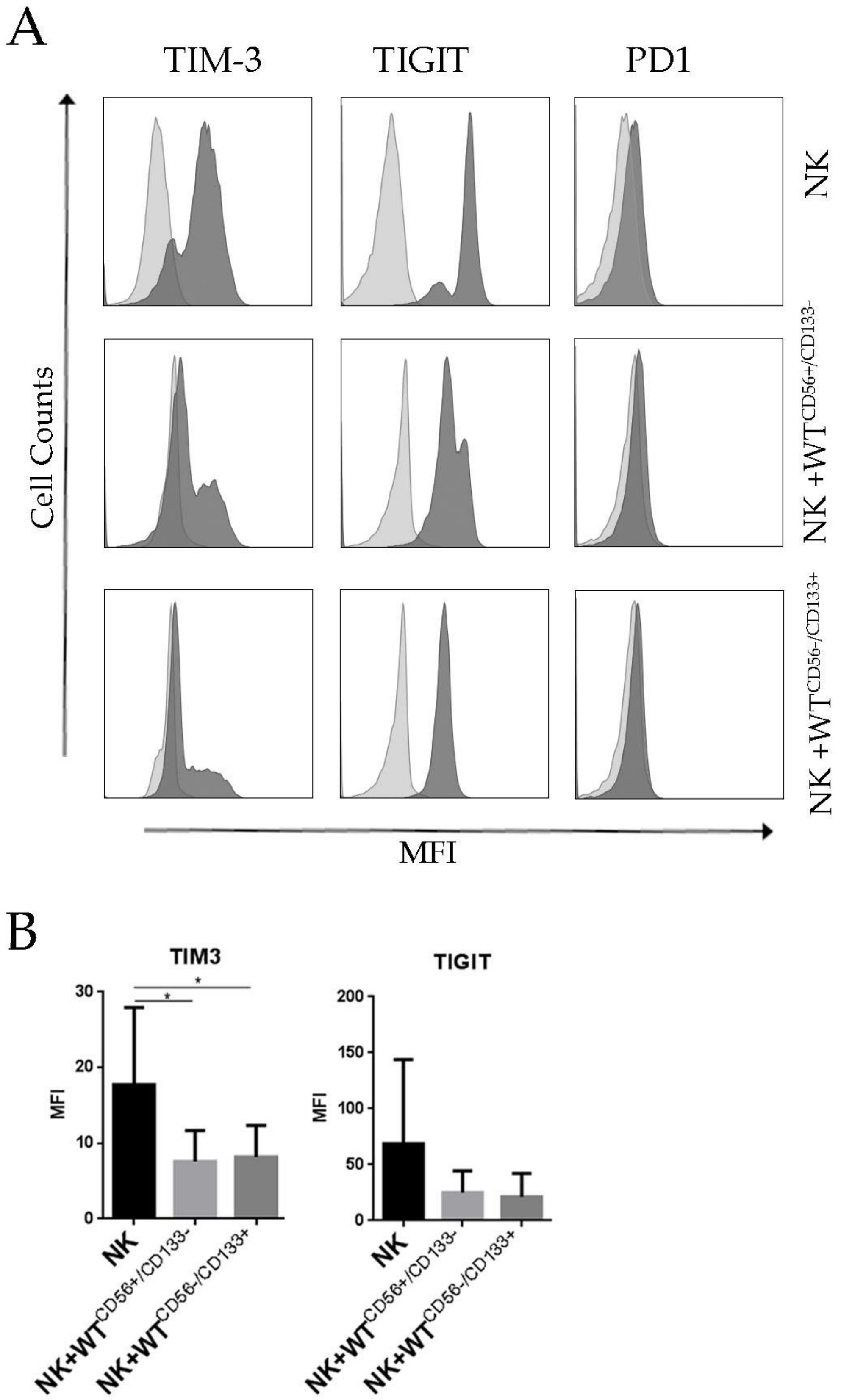

Figure S4. Analysis of check point molecules on NK cells by Flow Cytometry. (A). TIM-3, TIGIT and PD-1 surface expression on NK cells, co-cultured or not for six days with WT primary cultures were presented as histograms (One representative experiment out of 4 performed). (B) Bar graphs show the mean fluorescence

intensity (MFI) ratio between stained and unstained cells. The results are the means ±SD of four different experiments for each WT primary culture. P values were calculated using Mann Whitney test (*p<0.05).

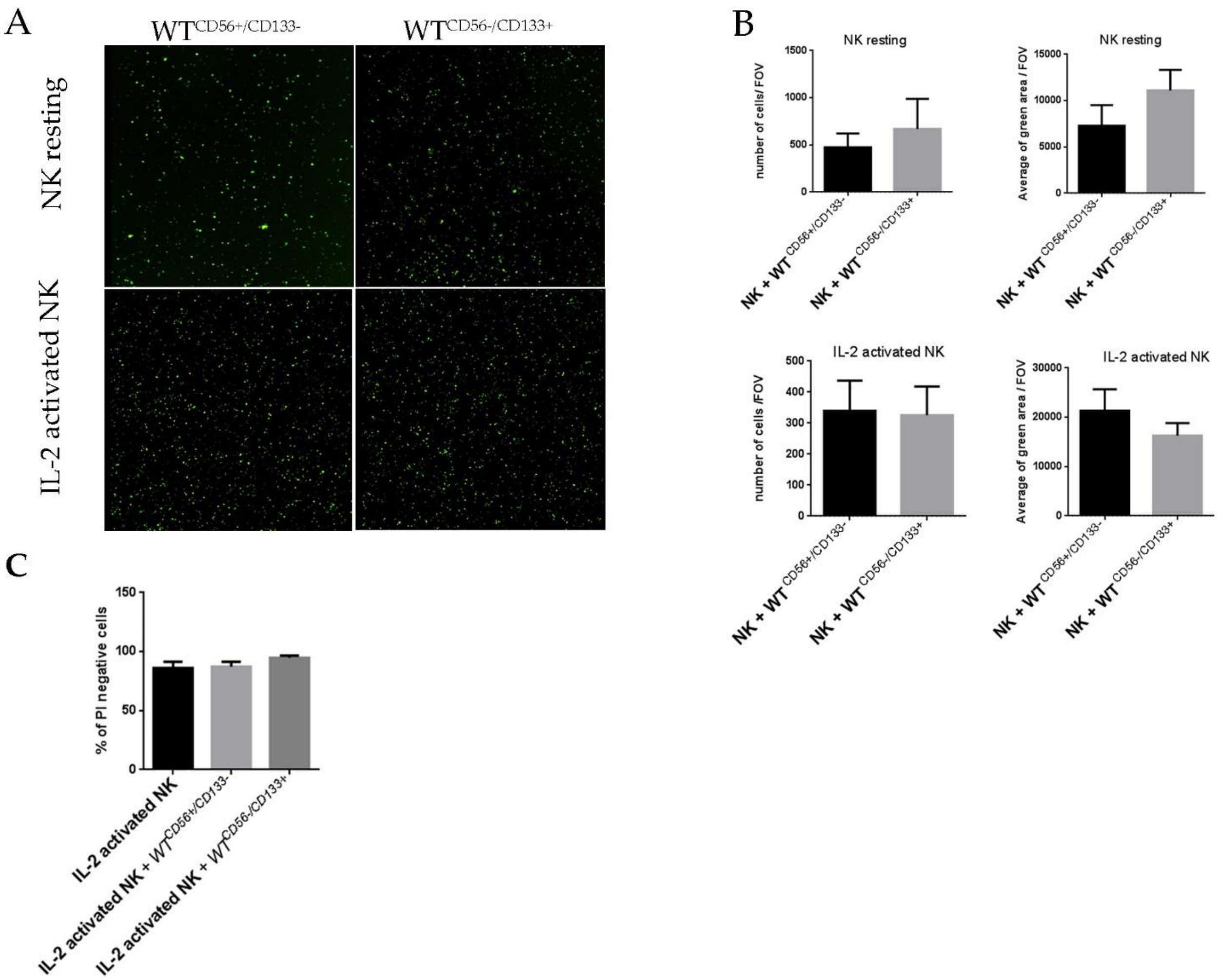

Figure S5. Binding of NK cells to different WT cell types. (**A**) Representative image provided from the Celigo S cytometer under green fluorescence. NK resting and IL-2 activated NK (green) were incubated with WT cells for two hours and, then, the not-adhered NK cells were removed with the medium. (**B**) The NK cells adhered to WT cells were reported as number of cell or total area of NK cells for field of view (FOV). Data shown are the average of three image from two independent experiments for each WT cell line ± SEM. Statistical significance was determined by t-test. (**C**) Percentage of Propidium Iodide (PI) negative cells ± SEM of 3 different IL2 activated NK cell donors after 6 days of incubation with WT cell line

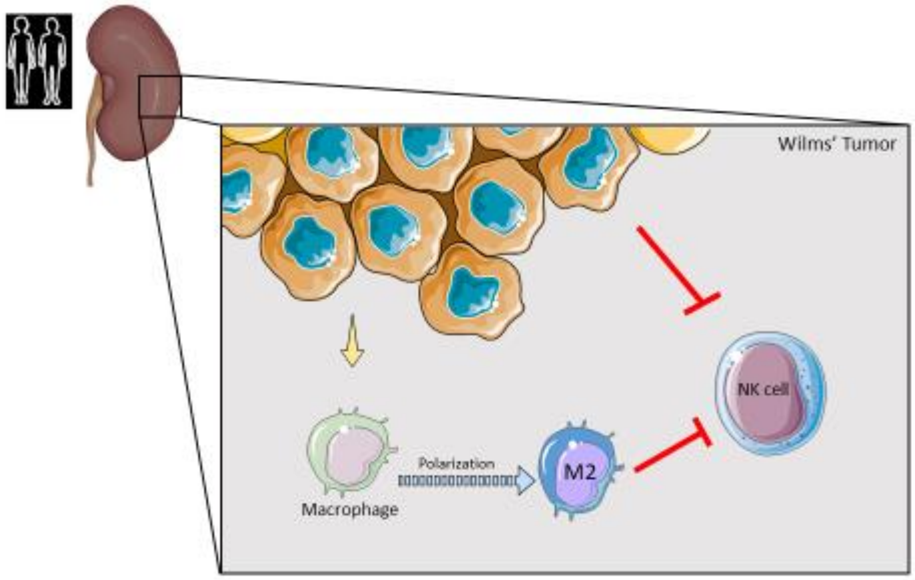

Figure S6 Scheme summarizing the interactions among WT cells, NK cells and or Macrophages
